# Supplementary material for: COVID-19 Vaccine Uptake and Associated Factors in Sub-Saharan Africa: Evidence from a Community-Based Survey in Tanzania
Source: Vaccines (Basel). 2023 Feb 17;11(2):465. doi: 10.3390/vaccines11020465 (PMC9961769; doi:10.3390/vaccines11020465)
Supplement: Supplementary file 1 [file vaccines-11-00465-s001.zip › vaccines-2100641-supplementary.pdf]

# Supplementary file S1: Questionnaire (English), community perceptions, concerns, and acceptability of COVID-19 vaccine in Tanzania

Dear Sir/Madam,
My name is ………………., a researcher from Kilimanjaro Christian Medical University College (KCMUCo).

You are hereby invited to participate in a study conducted by researchers from Kilimanjaro Christian Medical University College (KCMUCo), Muhimbili University of Health and Allied Sciences (MUHAS), St Francis University College of Health and Allied Sciences (SFUCHAS), and the National Institute for Medical Research (NIMR). The study aims to assess the knowledge, perceptions, COVID-19 vaccine acceptability, and associated factors among people in the communities in Tanzania". Understanding community perspectives and views on COVID-19 vaccine is key in shaping how the health providers will inform and educate the general public on covid-19 vaccines and which worry/ fears to address in a scientific manner. The MOHCDGEC would therefore like to capture early community perception and views in-order to offer targeted information where required and/or address common beliefs or myths.

The study follows strict guidelines for quality control, privacy and confidentiality. Your participation in this study will take approximately 20 minutes of your time and all information will be kept strictly confidential by the research team. No personal identifiers (such as your name, telephone number, or address) will be collected and your responses to the questionnaire will be anonymous. The information collected here (without any personal identifiers) will be put together into a technical report to assess knowledge and perceptions and identify barriers to COVID-19 vaccination in the country. Your participation is voluntary and please proceed if consented to participate in this study.

Do you agree to participate? Yes [  ] No [   ]

**I: Demographic and general Information of participants**

| Question | Response |
|---|---|
| 1. Region | *Put all 8 regions* |
| 2. District | *Auto populated as per the region* |
| 1. Ward name | |
| 2. Do you reside in an urban or rural area? | 1. Urban<br>2. Rural |
| 3. Date/ month and year of birth? | Date of birth (dd/mm/yyyy) |
| 4. What is your age in complete years | Age in complete years……… |
| 5. Sex | 1. Male<br>2. Female |
| 8. Highest level of education attained? completed? | Circle one<br>1. No formal education<br>2. Primary<br>3. Secondary (O-level)<br>4. Secondary (A-level)<br>5. Diploma<br>6. University<br>7. Others (specify……………….) |
| 9. What is your occupation that make your daily earning? | 1. Farming<br>2. Teaching<br>3. Business<br>4. Small scale business<br>5. Crafts or trade work<br>6. Services<br>7. Health work<br>8. Student<br>9. Not employed<br>10. Others (specify………………..) |
| 10. Marital status | 1.Married<br>2.Cohabiting<br>3.Single<br>4.Separated/divorced<br>5.Widow |
| 11. What is your religion? | 1.Muslim<br>2.Christian<br>3.No religion<br>4.Others (specify……………..…) |
| 12. Do you have health insurance? | 0.No<br>1.Yes |

**II: Household socio-economic status**

| | | |
|---|---|---|
| 13. Does this household own any livestock, herds, other farm animals, or poultry? | 0.No<br>1.Yes →13 | |
| 14. How many of the following animals does this household own?<br>IF NONE, RECORD '00'.<br>IF 95 OR MORE, RECORD '95'.<br>IF UNKNOWN, RECORD '98'. | | |
| a. Milk cows or bulls?<br>b. Other cattle?<br>c. Horses, donkeys, or mules?<br>d. Goats?<br>e. Sheep?<br>f. Chickens or other poultry? | ………..milk cows or bulls<br>………..other cattle<br>………..horses, donkeys, or mules<br>………..goats<br>………..sheep<br>………..chicken or other poultry | |
| 15. Does any member of this household own any agricultural land? | 0.No<br>1.Yes →16 | |
| 16. How many hectares of agricultural land do members of this household own?<br>IF 95 OR MORE, SELECT '950'.<br>IF DON'T KNOW, SELECT '998' | …………………………. hectares | |
| 17. What kind of toilet facility do members of your household usually use? | 1. Flush to piped sewage or septic tank<br>2. Pour flush latrine<br>3. Pit latrine (cement floor)<br>4. Pit latrine (mud floor)<br>5. No toilet | |
| 18. Where do you get your water? | 1. Tap water (piped through house or at yard)<br>2. Public standpipe<br>3. Protected tube well or bore hole<br>4. Spring<br>5.Rainwater (cistern or inter tank)<br>6. Pond-water or stream<br>7. Other specify………………… | |
| 19. Where is cooking usually done? | 1. In a room used for living or sleeping<br>2. In a separate room used as kitchen<br>3. In a separate building used as kitchen<br>4. Outdoors | |
| 20. Does your household have: | Yes | No |
| a. Electricity that is connected? | | |
| b. A radio in working condition? | | |
| c. A television in working condition? | | |
| d. A computer in working conditions? | | |
| e. A refrigerator in working condition? | | |

| | | |
|---|---|---|
| f. A battery or Generator for power? | | |
| g. An iron (charcoal or electricity) | | |
| 21. Does any member of this household own: | Yes | No |
| a. A watch | | |
| b. A mobile phone | | |
| c. A bicycle | | |
| d. A motorcycle or motor scooter? | | |
| e. An animal-drawn cart? | | |
| f. A car or truck | | |
| g. A boat with a motor? | | |
| 22. **OBSERVE:** Type of floor | Yes | No |
| a. Hard Floor (tile, cement, concrete, brick, wood) | | |
| b. Earth/sand | | |
| c. Dung | | |
| d. Other, specify ………… | | |
| 23. **OBSERVE:** Type of Wall | Yes | No |
| a. Cement, brick, or stone | | |
| b. Grass | | |
| c. Cane, palm, trunks, bamboo | | |
| d. Mud, poles with mud | | |
| e. Thatched/ grass | | |
| f. Metal sheet | | |
| g. Plastic sheet | | |
| h. Others, specify ………… | | |
| 24. **OBSERVE:** Type of roof | Yes | No |
| a. Grass/thatch/palm leaf/mud | | |
| b. Rustic mat | | |
| c. Palm/bamboo | | |
| d. Wood planks | | |
| e. Iron sheet | | |
| f. Concrete | | |
| g. Tiles | | |
| h. Others, specify ………… | | |
| 25. Do you own a bank account? | | |
| | | |

**III: Awareness and knowledge on covid-19 disease**

| | |
|---|---|
| 25. Do you think COVID-19exists in Tanzania? | 0. No<br>1. Yes<br>2. I do not know |
| 26. How do you perceive your risk for getting COVID-19 infection? | 1. Not at all<br>2. Very low risk<br>3. Low risk<br>4. Medium risk<br>5. High risk |

| | |
|---|---|
| | 6. Very high risk<br>7. I do not know |
| 27. Have you ever been infected with COVID-19? | 0. No → to Q 29<br>1. Yes<br>2. I don't know → to Q 29 |
| 28. If Yes, how did you came into conclusion that you were infected? | 1. I was tested and received positive results<br>2. Told by doctor from the symptoms<br>3. I had symptoms and concluded I had covid<br>4. I came in contact with a person infected with covid 19 and I got similar symptoms<br>5. I was told by a relative/ neighbor due to my symptoms<br>6. I attended burial<br>7. Others (please mention……….) |
| 29. Has any of your family member (s) been infected with COVID-19? | 0. No<br>1. Yes |
| 30. Has any of your family members died from COVID-19? | 0. No<br>1. Yes |
| 31. Has any of your close friend(s) died from COVID-19? | 0. No<br>1. Yes |

**IV: Awareness and knowledge on covid-19 vaccines**

| | |
|---|---|
| 32. Have you heard about COVID-19 vaccine? | 0. No<br>1. Yes<br>2. I don't know |
| 33. Which vaccines have you heard about? mention them | *Interviewer do not read response select/tick all mentioned*<br>1. Johnson and Johnson's (Jansen)<br>2. Sinopharm<br>3. Sinovac<br>4. Moderna<br>5. Pfizer-BioNTech<br>6. AstraZeneca/ Covishield<br>7. Sputnik V<br>8. Novavax<br>9. Others (specify…………….....) |
| *34.* What is the recommended age to give COVID-19 vaccine in Tanzania? | *……………………age*<br>*If don't know enter 999* |
| 35. Who has been prioritized to receive vaccination in Tanzania? | *Circle/Tick all mentioned*<br>1. Anybody aged 18 years and above<br>2. Health care providers<br>3. Older people (50 and above) |

| | | | |
|---|---|---|---|
| | 4. People with comorbidities (DM, hypertension, CVD, asthma, chronic respiratory disease)<br>4. Others (specify………………..) | | |
| 36. Regarding COVID-19 vaccine | | | |
| a. Can a person get infected by vaccination? | 0. No | 1. Yes | 2. I don't know |
| b. Can vaccinated person get infected with Covid-19? | 0. No | 1. Yes | 2. I don't know |
| c. COVID-19 vaccine can be given to people who had been infected/ sick with COVID-19? | 0. No | 1. Yes | 2. I don't know |
| d. A person who has received COVID-19 vaccine needs to continue following traditional preventive methods (e.g. distancing, handwashing, use of mask) | 0. No | 1. Yes | 2. I don't know |
| e. COVID-19 vaccines can reduce the risk/ chance of being infected with COVID-19? | 0. No | 1. Yes | 2. I don't know |
| f. COVID-19 vaccines prevent severe infection? | 0. No | 1. Yes | 2. I don't know |
| g. COVID-19 vaccines reduce deaths due to COVID-19? | 0. No | 1. Yes | 2. I don't know |
| 37. Who should not be given COVID-19 vaccine? | *Interviewer record all responses mentioned spontaneously after reading the question* | | |
| A person with history of allergy to other vaccines | 0. No | 1. Yes | 2. Don't know |
| A person with high fever on the day of vaccination (> $38.5^{O}$c) | 0. No | 1. Yes | 2. Don't know |
| A pregnant woman | 0. No | 1. Yes | 2. Don't know |
| A person who had been infected with COVID-19 before | 0. No | 1. Yes | 2. Don't know |
| A person who have COVID-19 symptoms on the day of vaccination | 0. No | 1. Yes | 2. Don't know |
| Others (specify……………………) | | | |

**V: Perceptions, views, and practice on Covid-19 vaccines**
***We will now talk about your perceptions and views on COVID-19 vaccine.***

***For Q 38, interviewer, please orient the participant the question answers is in Likert scale from strongly agree, agree, neither, disagree or strongly disagree***

| 38. Regarding your perceptions and views on covid-19 vaccines | Strongly agree | Agree | Neither | Disagree | Strongly disagree |
|---|---|---|---|---|---|
| a. Covid-19 vaccines work | | | | | |
| b. Covid-19 vaccines are safe for use in humans | | | | | |
| c. Standard vaccines are safer than Covid-19 vaccine | | | | | |
| d. Covid-19 vaccines have passed rigorous quality control for use | | | | | |
| e. In general, benefits of COVID-19 vaccines outweigh the risks. | | | | | |
| f. I am concerned that a shot might not prevent the disease | | | | | |
| g. Shortcuts which are taken to develop and approve vaccines during a pandemic emergency compromise their safety. | | | | | |
| h. I trust that the Ministry of Health has ensured that the vaccines made available in Tanzania are safe. | | | | | |
| i. My concerns about related to side effects (minor/ short term effects) prevents me from taking COVID-19 vaccines. | | | | | |
| j. My concerns about adverse events (big side effects) prevents me from taking COVID-19 vaccines | | | | | |
| k. The government is pushing you into a vaccination decision you do not fully support | | | | | |

| | |
|---|---|
| 39. Have you taken the Covid-19 vaccine? | 1. Have already taken the vaccine →Q 40<br>2. Will wait for sometimes →Q 42<br>3. Will not take the vaccine at all →Q 42 |
| 40. If you have taken, what was the month /year taken? | dd/mm/yyyy |
| 41. If you have taken, which vaccine have you taken and how many times? | Name (s):………………<br>Number of doses……… |

*42.* If you will delay or not take at all why? *(Interviewer circle all that have been mentioned)*

*1.* This is a new vaccine (too new)

| | |
|---|---|
| *2.* Not enough time to develop it<br>*3.* Concerned/fear of vaccine side effects<br>*4.* I think the vaccine is not effective (concerned about efficacy)<br>*5.* I think the vaccine is designed to harm us (concerned about safety)<br>*6.* My body is naturally strong, I don't need a vaccine to fight COVID-19<br>*7.* I already had COVID-19 so I think I am immune to the disease<br>*8.* The COVID-19 pandemic is finished in my country, no need for a vaccine now<br>9. I do not like needles<br>10. I need more time to make this decision<br>11. I prefer others to get vaccinated first<br>12. My partner does not approve that I should get vaccinated<br>13. My friends/ co-workers do not approve that I should get vaccinated<br>14. My religious leaders do not approve the vaccine<br>15. My local (organisational, political, cultural) leaders do not approve the vaccine<br>16. The vaccine is not available in the facility I work / nearby facility<br>17. I lack information regarding the vaccine<br>18. I do not trust pharmaceutical companies<br>19. I have medical contraindications to the vaccine<br>20. Prefer to develop natural immunity by getting COVID-19<br>21. I do not think the vaccine will protect me<br>22. Free vaccines are not good<br>23. Others (specify) | |
| 43. If given a chance to select COVID-19 vaccine, which of the following COVID-19 vaccine would you prefer to use? | *Interviewers select all that are mentioned*<br>1. Pfizer-BioNTech<br>2. Moderna<br>3. Johnson and Johnson's (Jansen)<br>4. AstraZeneca/ Covishield<br>5. Sinovac<br>6. Sinopharm<br>7. Sputnik V<br>8. Sputnik Light<br>9. Novavax<br>10. Oxford<br>11. None of the above<br>12. I don't know the vaccines<br>13. Others (specify………………..) |
| 44. Do you have any concerns or worries regarding the Covid-19 vaccine that are being used now in the country? | 0. No<br>1. Yes<br>2. Don't know |
| If Yes, what are your concerns or worriers regarding the COVID-19 vaccines that are being used now in the country? | |

| | |
|---|---|
| 45. Are you worried that some people in your community who are delaying or refusing vaccines, are putting you at risk? | 0. No<br>1. Yes<br>2. I don't know |

**VI: Source of information and its influence on COVID-19 uptake**

***We will now talk about your source of COVID-19 vaccine information and its influence***

| | |
|---|---|
| 46. Which source of information on COVID-19 vaccine do you know? Please mention all the sources (*Interviewer tick all that will be mentioned)* | *Interviewer do not read, circle or tick all mentioned*<br>1. Healthcare providers<br>2. The ministry of health<br>3. The government<br>4. Religious leaders<br>5. Local leaders (clan or cultural)<br>6. Local leaders (political/ politicians)<br>7. Family/ friends<br>8. My partner<br>9. Media like television, radio, magazines<br>10. Social media like *Instagram, Whatsapp, Twitter, Facebook*<br>11. World Health Organization (WHO)<br>12. Scientific papers/ reports (articles/ journals etc)<br>13. Others (specify……………………………….…) |
| 47. Which source of information on COVID-19 vaccine do **you trust**? | *Interviewer do not read, circle or tick all mentioned*<br>1. Healthcare providers<br>2. The ministry of health<br>3. The government<br>4. Religious leaders<br>5. Local leaders (clan or cultural)<br>6. Local leaders (political/ politicians)<br>7. Family/ friends<br>8. My partner<br>9. Media like television, radio, magazines<br>10. Social media like Instagram, Whatsapp, Twitter, Facebook<br>11. World Health Organization (WHO)<br>12. Scientific papers/ reports (articles/ journals etc)<br>13. Others (specify……………………………….…) |
| 48. Which source(s) of information on COVID-19 give **false/wrong** information on COVID-19 vaccines? | *Interviewer do not read, circle or tick all mentioned*<br>1. Healthcare providers<br>2. The ministry of health<br>3. The government<br>4. Religious leaders<br>5. Local leaders (clan or cultural)<br>6. Local leaders (political/ politicians)<br>7. Family/ friends |

| | |
|---|---|
| | 8. My partner<br>9. Media like television, radio, magazines<br>10. Social media like Instagram, Whatsapp, Twitter, Facebook<br>11. World Health Organization (WHO)<br>12. Scientific papers/ reports (articles/ journals etc)<br>13. Others (specify…………………………….….) |
| 49. Have reports you heard/read in the media/ on social media made you reconsider or refuse to take Covid-19 vaccine? | 0. No<br>1. Yes |
| 50. Do you think reports that people hear and/or read in the media or social media influenced your friends, relatives or family members to refuse COVID-19 vaccine? | 0. No<br>1. Yes |
| 51. Do you share information related to covid-19 vaccination within your social media network? | 0. I do not share<br>1. I share |
| 52. Covid-19 vaccine has been widely debated in the media/ and in social media platform. In your opinion, do you think this debate is good or bad? | 1. Good<br>2. Bad |

**VII: Influence of leaders (*community leaders, religious leaders, celebrities*) on COVID-19 vaccine acceptance or hesitancy**

| | |
|---|---|
| 53. Do religious leaders in your community support COVID-19 vaccines? | 0. No<br>1. Yes<br>2. I don't know |
| 54. Do teachers in your community support COVID-19 vaccines? | 0. No<br>1. Yes<br>2. I don't know |
| 55. Do health workers leaders in your community support COVID-19? | 0. No<br>1. Yes<br>2. I don't know |
| 56. Do political leaders in your community support COVID-19 vaccines? | 0. No<br>1. Yes<br>*2.* I don't know |
| 57. Has your religious leader ever advocated against COVID-19 vaccination? | 0. No<br>1. Yes |
| 58. Has the teachers in your community ever advocated against COVID-19 vaccination? | 0. No<br>1. Yes |

| | | |
|---|---|---|
| 59. | Has health workers in your community ever advocated against COVID-19 vaccination? | 0. No<br>1. Yes |
| 60. | Has political leaders in your community ever advocated against COVID-19 vaccination? | 0. No<br>1. Yes |

**VIII: Influence of politics/ policies on COVID-19 vaccine acceptance or hesitancy**

| | |
|---|---|
| 61. Do you trust that the government Is making decisions in your best interest with respect to what the covid-19 vaccines are provided? | 0. No<br>1. Yes |

**IX: Geographical barriers and COVID-19 vaccine uptake**

| | |
|---|---|
| 62. Do you know where to get COVID-19 vaccine?<br>Mention where?................. | |
| 63. Does distance to vaccination centers prevents you from getting yourself vaccinated for COVID-19? | 0. No<br>1. Yes |
| 64. Does timing of covid-19 vaccination at vaccination centers prevents you from getting yourself vaccinated for COVID-19? | 0. No<br>1. Yes |
| 65. Does waiting time at the vaccination centers prevents you from getting yourself vaccinated for COVID-19? | 0. No<br>1. Yes |
| 66. Does cost in getting to the vaccination centers prevent you from getting yourself vaccinated for COVID-19? | 0. No<br>1. Yes |

**WE HAVE COME TO THE END OF INTERVIW**

| |
|---|
| Interviewers record any important issue that you have observed during the interview that were not captured in the questionnaire |
